# Computational Modeling and Analysis of Iron Release from Macrophages

**DOI:** 10.1371/journal.pcbi.1003701

**Published:** 2014-07-03

**Authors:** Alka A. Potdar, Joydeep Sarkar, Nupur K. Das, Paroma Ghosh, Miklos Gratzl, Paul L. Fox, Gerald M. Saidel

**Affiliations:** 1 Department of Biomedical Engineering, Case Western Reserve University, Cleveland, Ohio, United States of America; 2 Department of Cellular and Molecular Medicine, Cleveland Clinic, Cleveland, Ohio, United States of America; Bayer Technology Services GmbH, Germany

## Abstract

A major process of iron homeostasis in whole-body iron metabolism is the release of iron from the macrophages of the reticuloendothelial system. Macrophages recognize and phagocytose senescent or damaged erythrocytes. Then, they process the heme iron, which is returned to the circulation for reutilization by red blood cell precursors during erythropoiesis. The amount of iron released, compared to the amount shunted for storage as ferritin, is greater during iron deficiency. A currently accepted model of iron release assumes a passive-gradient with free diffusion of intracellular labile iron (Fe^2+^) through ferroportin (FPN), the transporter on the plasma membrane. Outside the cell, a multi-copper ferroxidase, ceruloplasmin (Cp), oxidizes ferrous to ferric ion. Apo-transferrin (Tf), the primary carrier of soluble iron in the plasma, binds ferric ion to form mono-ferric and di-ferric transferrin. According to the passive-gradient model, the removal of ferrous ion from the site of release sustains the gradient that maintains the iron release. Subcellular localization of FPN, however, indicates that the role of FPN may be more complex. By experiments and mathematical modeling, we have investigated the detailed mechanism of iron release from macrophages focusing on the roles of the Cp, FPN and apo-Tf. The passive-gradient model is quantitatively analyzed using a mathematical model for the first time. A comparison of experimental data with model simulations shows that the passive-gradient model cannot explain macrophage iron release. However, a facilitated-transport model associated with FPN can explain the iron release mechanism. According to the facilitated-transport model, intracellular FPN carries labile iron to the macrophage membrane. Extracellular Cp accelerates the oxidation of ferrous ion bound to FPN. Apo-Tf in the extracellular environment binds to the oxidized ferrous ion, completing the release process. Facilitated-transport model can correctly predict cellular iron efflux and is essential for physiologically relevant whole-body model of iron metabolism.

## Author Summary

Iron metabolism is an important physiological phenomenon essential for sustaining life. There is a tight regulation of iron levels in humans and both deficiency and overload can lead to disorders such as anemia and hemochromatosis. Recycling of iron in human body via macrophage iron release is crucial to maintain healthy iron levels. However, a computational model is needed to quantitatively analyze the mechanism underlying a key process in iron homeostasis, which is the release of iron from the macrophages. Using mechanistic, mathematical models to simulate experimental data, we found a novel mechanism by which macrophages release iron. A comparison of experimental data with model simulations shows that a currently accepted passive-gradient mechanism cannot represent the iron-release process from macrophages. However, our model with a facilitated-transport mechanism associated with ferroportin (only known protein for iron export) accurately reproduces the iron release process. This model quantifies for the first time the detailed molecular mechanism associated with iron transport via ferroportin. This quantitative predictive model of cellular iron efflux is essential for physiologically relevant simulation of whole-body model of iron metabolism in healthy and disease states.

## Introduction

The body maintains strict control of iron levels to avoid both iron deficiency and excess. One of the major processes of iron homeostasis in whole-body iron metabolism is the release of iron from the macrophages of the reticuloendothelial system. In adult men, iron absorption in the small intestine is about 1 mg/day, iron loss is about 1 mg/day, and iron recycling from macrophages into plasma and then into erythrocytes is about 25 mg/day [1]. Iron efflux is tightly controlled by the plasma iron requirement. In several pathological states including chronic inflammation and renal failure, macrophages accumulate iron because of an apparent inability to release iron normally.

From a study of intestinal iron absorption in perfused rat liver, data were consistent with the concept of passive iron efflux across the intestinal epithelium [2]. A negative iron gradient across the serosal wall of the epithelial cells was presumed to cause an iron efflux. Intracellular iron is readily available for release from the labile iron pool (LIP), which is mostly in the ferrous state [3], and from the sequestered iron in the ferric state. Ferrous iron in the labile iron pool is either sequestered to iron storage in ferritin (in ferric form) or transported across the cell membrane (in ferrous form) into plasma [4]. At the cell membrane, the ferrous form becomes oxidized to ferric. According to this passive-gradient mechanism, iron is transported across the cell membrane in the ferrous form, which is a more usable (labile) form in cells. To maintain the iron gradient, apo-transferrin (Tf), the primary carrier of soluble iron in the plasma, binds and removes iron from the site of efflux.

A key protein involved in macrophage iron efflux is ceruloplasmin (Cp). This plasma protein Cp has ferroxidase activity, which is critical in iron efflux as shown by Osaki and Johnson [5]. They hypothesized that Cp accelerates oxidation and binding of iron to apo-Tf. Consequently, the negative iron gradient is increased across the cellular membrane that produces an iron efflux. Cp protein level is known to be regulated by a cis-regulatory GAIT (interferon-gamma activated inhibitor of translation complex) element [6]. Binding to GAIT complex leads to translational silencing of Cp transcript to some minimal basal level [7]. The *in vivo* role of Cp in iron homeostasis is evident from iron overload in patients with aceruloplasminemia (i.e., hereditary Cp deficiency) [8].

The effect of Cp as a plasma ferroxidase in iron release depends on other factors. Young et al. [9] reported a 40% stimulation of iron release from human hepatocarcinoma HepG2 cells by Cp in the presence of apo-Tf. Also, they reported that low oxygen concentration stimulated iron release by Cp. Furthermore, Cp stimulated iron release from macrophages in the presence of apo-Tf and hypoxia with a large LIP [10]. In a dose-dependent manner, Cp enhanced iron release in HepG2 cells compared to bovine serum albumin (BSA) control [11].

Another factor that affects macrophage iron release is ferroportin (FPN), an iron transporter [12], [13]. FPN is essential for intestinal iron absorption as shown by knock-out mice studies [13]. The role of FPN in iron release has been demonstrated in several cell types [14], [15]. The specific role of FPN in up-regulation of iron release from macrophages after erythrophagocytosis has been examined [16]. The passive-gradient mechanism assumes that FPN allows free diffusion of iron across the plasma membrane. In macrophages, however, FPN is apparently distributed throughout the cell and not predominantly on the plasma membrane [17], which raises uncertainty about its proposed role in passive-gradient mechanism of iron release. Another study has shown that ferroxidases such as Cp can maintain cellular iron efflux by stabilizing FPN on cell membrane [18].

**Figure 1:**
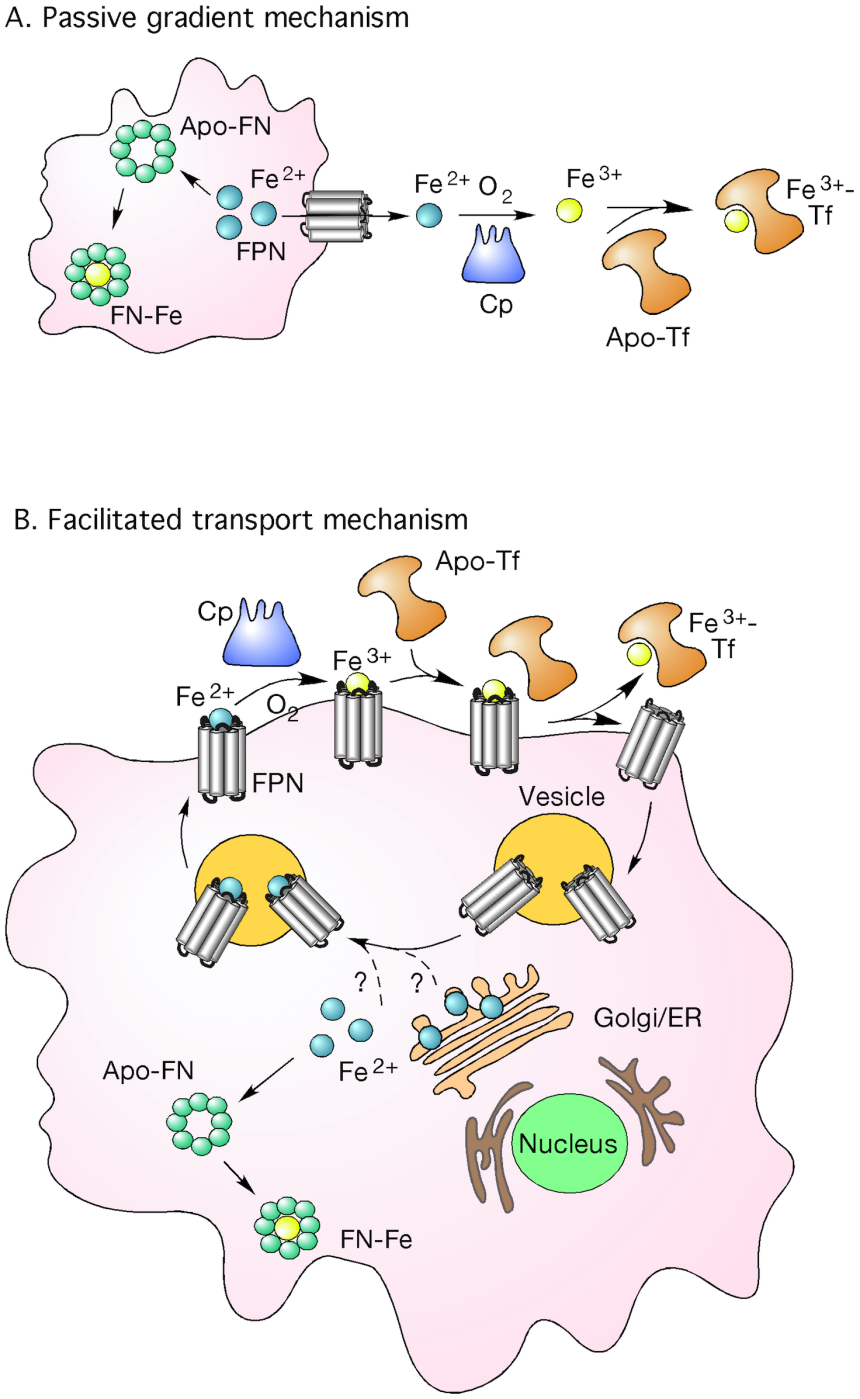
Cartoon of facilitated-transport and passive-diffusion mechanisms for macrophage cell iron efflux. Fig 1A: Passive-diffusion mechanism where negative ferrous iron gradient across the cell membrane drives the cellular iron release. Fig 1B: Facilitated-transport mechanism where FPN plays an active role and carries ferrous iron to cell membrane for cellular iron release.

The passive-gradient mechanism has been a dominant concept in macrophage iron release, but it has not been quantitatively analyzed by a mathematical model that includes detailed kinetic processes. Although FPN is associated with the iron release process, the molecular mechanism for iron transport from macrophages via FPN has not been established [8]. In this study, we hypothesized that iron efflux involves a facilitated-transport process in which FPN binds the ferrous iron and carries it to the cellular membrane for iron release. To distinguish the effects of passive and facilitated-transport mechanisms, we compare simulations of mathematical models to experimental data. These mechanisms are represented in the cartoons shown in Fig. 1. The models that describe the detailed transport and kinetic processes of iron release are based on dynamic mass balances for each of the molecular species in intracellular, membrane, and extracellular domains. The computational outputs of these models are compared to experimental data from studies of iron kinetics in solution and from *in vitro* studies of iron release from macrophages. Consequently, the validity of the passive and facilitated-transport hypotheses can be quantitatively ascertained. The new facilitated-transport model could accurately reproduce the iron release process and describe for the first time the detailed molecular mechanism associated with iron transport via FPN.

## Results

### Mathematical Models

In this section, we develop mathematical models of iron kinetics and transport to quantify mechanisms associated with iron release from macrophages. The major issue is whether a passive-gradient or facilitated-transport mechanism is most likely. The simplest model with the passive-gradient mechanism assumes spatially uniform (or lumped) solute concentrations. To represent the *in vitro* experiments with a macrophage culture, a model must incorporate an extracellular (EC) domain with spatially distributed solute concentrations. Such a spatially distributed domain is included in passive-gradient (spatially distributed form) and facilitated-transport models.

The passive-gradient models assume that FPN produces selective permeability of ferrous form of iron so that ferrous ion can diffuse from the cell. According to this model, high intracellular concentration of ferrous ion across the cell produces a concentration gradient that causes passive diffusion of ferrous ion. In the facilitated-transport model, ferrous ion must be bound to FPN in order to be transported out of the cell. In this model, FPN species kinetics must be included because FPN does not merely act as a passive channel.

Regardless of the model used to describe iron transport and kinetics in cell culture, some basic aspects of iron kinetics can be analyzed from studies in solution. This information is applicable for analysis of iron kinetics in the EC domain of the cell culture. In the passive-gradient and facilitated-transport models, some common mathematical aspects are presented before examining the distinctions between these models.

#### Iron kinetics in solution

**Figure 2:**
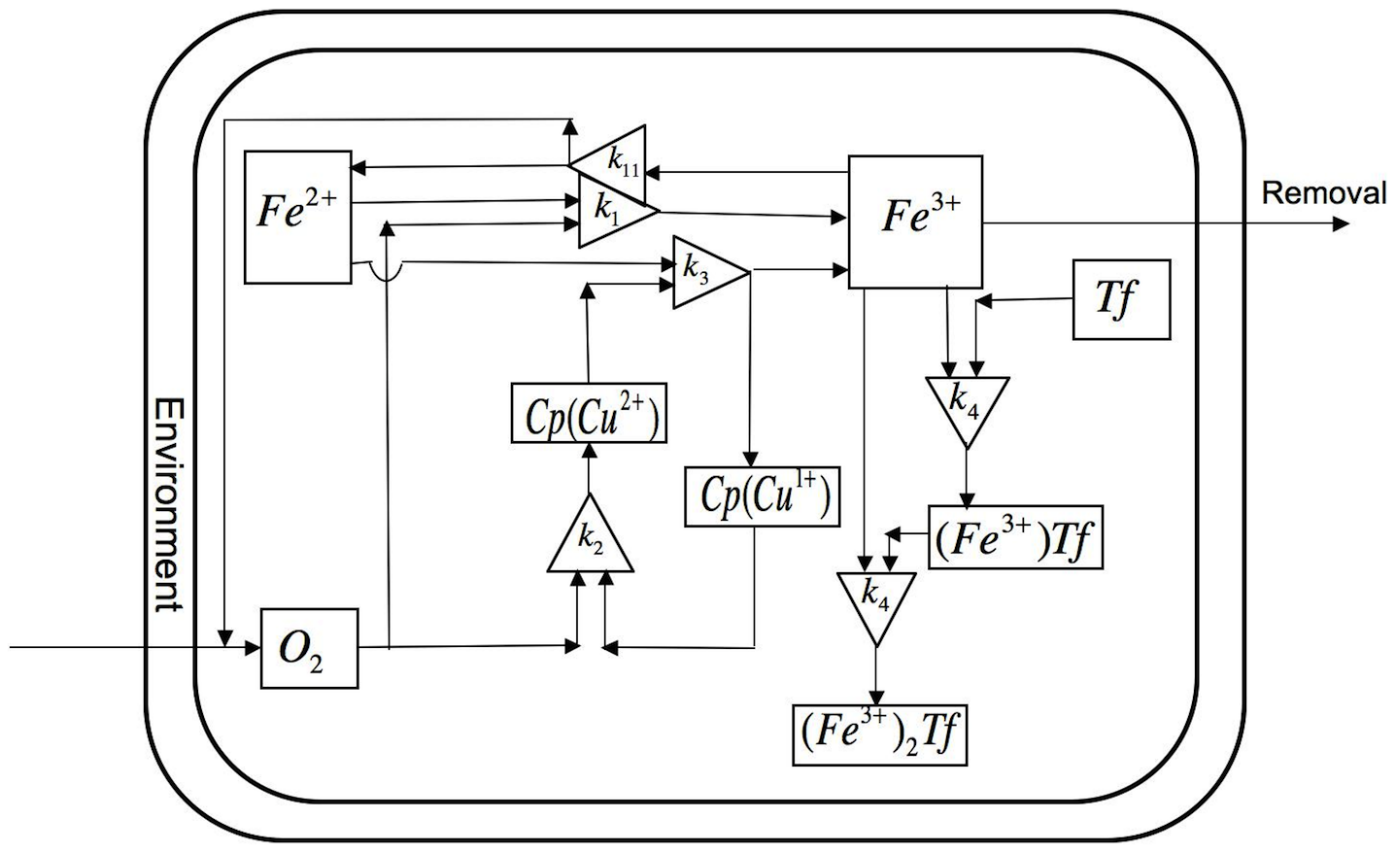
Schematic representation of iron kinetics in solution. Reactions when ferrous ion is added to a solution containing Cp and apo-Tf. Ferrous ion is oxidized by molecular O_2_ and ferroxidase Cp. Ferric ion binds to apo-Tf to form mono-ferric ((Fe^3+^)Tf) and di-ferric transferrin ((Fe^3+^)_2_Tf).



Key aspects of iron kinetics that can be studied in solution are shown in (Fig. 2). The diagram for the solution kinetics model using systems biology graphical notation (SBGN) is shown in Fig. S1. These include reversible oxidation of ferrous iron (Fe^2+^) by oxygen to ferric iron (Fe^3+^) dependent on pH:

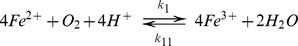
(K.1)Oxidation of ferrous iron by (oxidized) ceruloplasmin Cp(Cu^2+^):
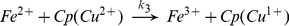
(K.2)


Oxidation of (reduced) ceruloplasmin Cp(Cu^1+^):
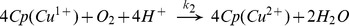
(K.3)


Binding of *Fe^3+^* to apo-transferrin:
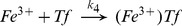
(K.4)


Ferric iron binds to transferrin very strongly and the off-rate for the reverse reaction is very small so that we can assume that the binding is irreversible. The affinity constant for binding of ferric iron to transferrin binding sites is very high (10^22^M^−1^) [19].

Binding of *Fe^3+^* to mono-ferric transferrin (*Fe^3+^)Tf* to form holo-Tf or diferric transferrin 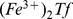:
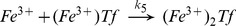
(K.5)


In order to simulate iron kinetics in solution, we include an additional reaction to account for ferric ion loss, which may occur by precipitation:
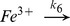
(K.6)


For a closed system in solution, we construct the dynamic mass balance of each molecular species corresponding to the subscript 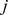  = 1,2,…8 (see Table 1). The species concentration *X_j_(t)* changes according to the reaction rate per unit volume 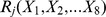:

**Table 1:** Reaction equations for iron kinetics in solution.

Species 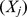	Specific reaction rate 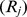
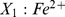	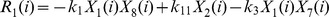
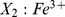	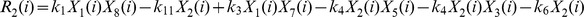
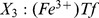	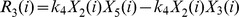
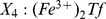	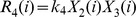
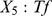	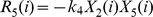
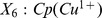	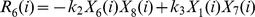
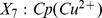	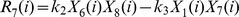
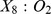	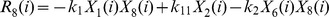



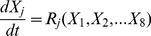
(1)


As shown in Table 1, the reaction rates depend on the solute concentration kinetics.

The solute concentrations at any time are related to initial concentrations through the conservation of total mass *M_s_* for iron (s = Fe), ceruloplasmin (s = Cp) and transferrin (s = Tf) in all their forms: 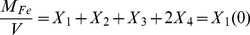
(2)




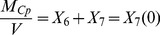
(3)




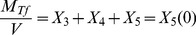
(4)where the solution volume *V* is constant.

#### Iron transport and kinetics in cell culture

**Figure 3:**
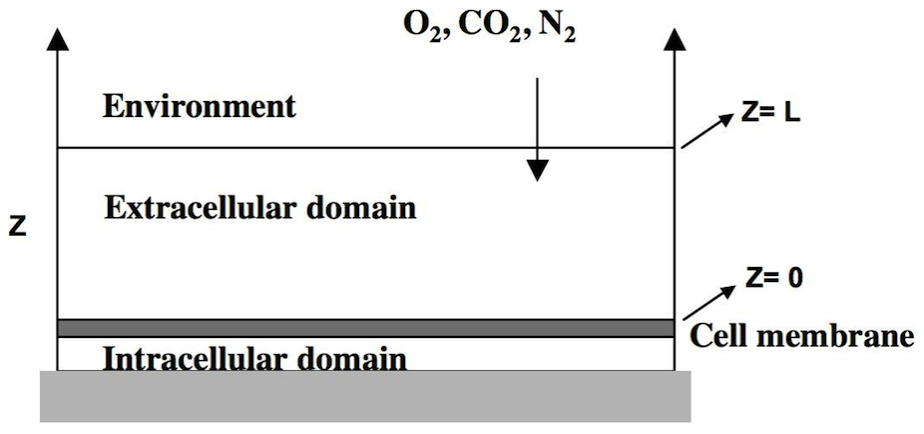
Experimental cellular iron release system. Experimental system consists of a monolayer of cells in a culture dish. Extracellular fluid in contact with fixed O_2_ and CO_2_ gas fractions.

**Figure 4:**
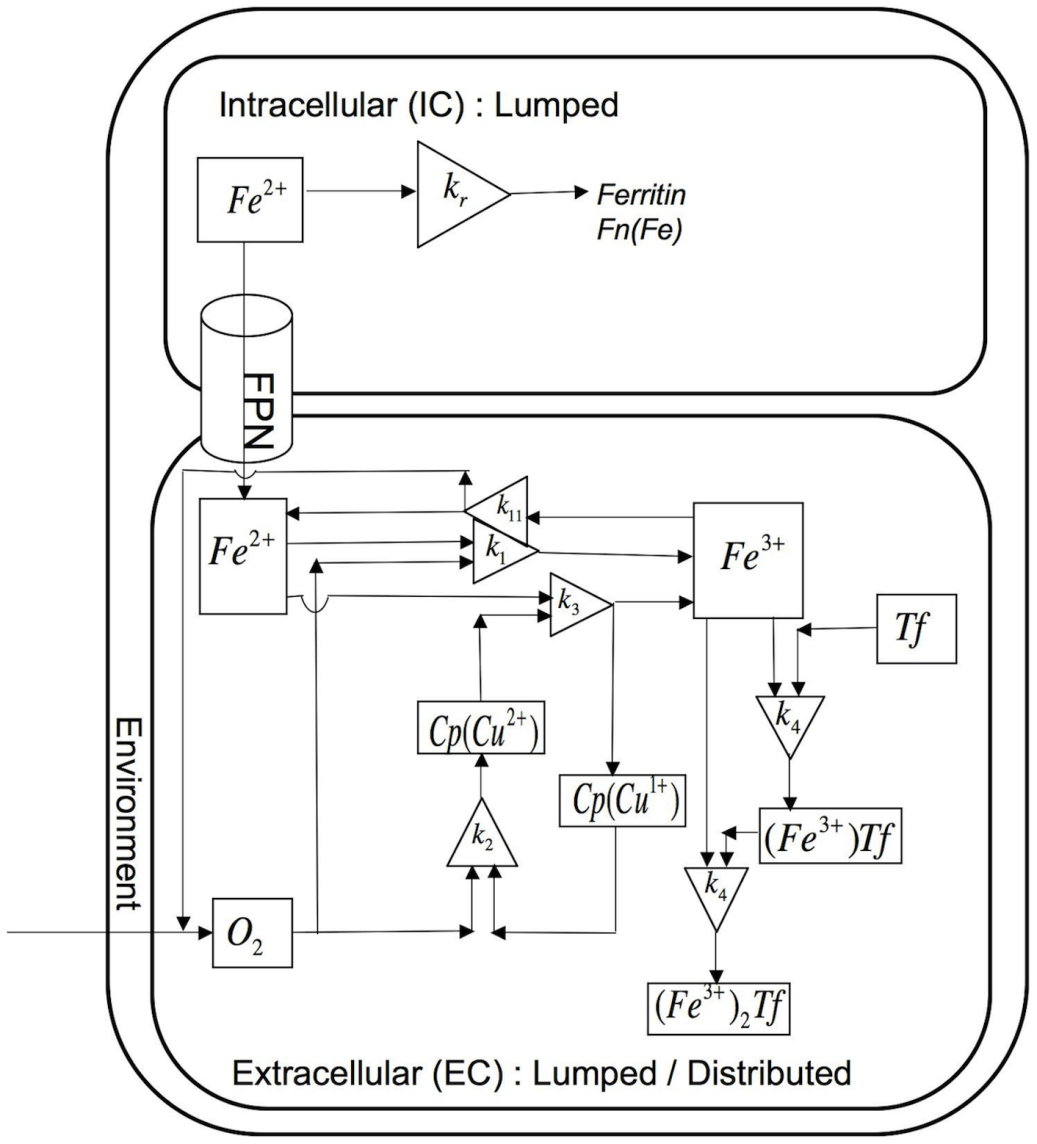
Schematic representation of the passive-gradient models: Spatially lumped (SL) and spatially distributed (SD). In the SL passive-gradient model, iron is released from the cells to a SL extracellular domain. In the SD gradient model, iron is released from the cells to a SD extracellular domain. The driving force for iron release in passive-gradient models is a concentration gradient of ferrous iron across the plasma membrane. Ferroportin acts as a selective channel through which iron diffuses passively in both the passive-gradient models. Reactions in the EC fluid are identical to those of iron kinetics in solution in both versions of the model.

**Figure 5:**
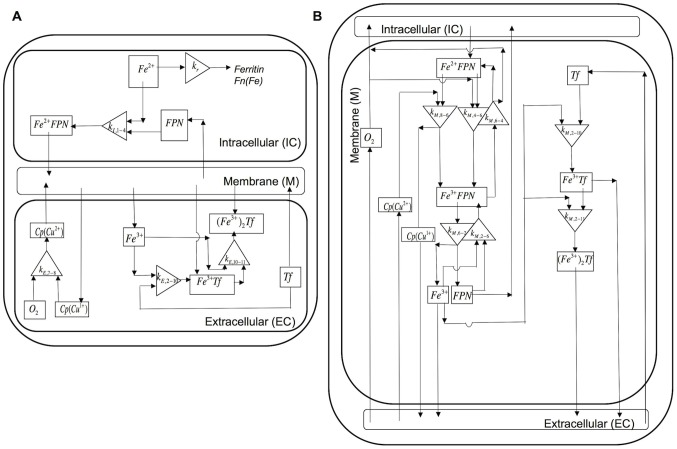
Schematic representation of the facilitated-transport model. Fig 5A: Reaction processes in intracellular and extracellular domain. Intracellular and membrane domains are spatially lumped. Extracellular domain is spatially distributed. Fig 5B: Reaction processes in membrane domain. Ferroportin carries ferrous ion to the plasma membrane. Outside the cell, Cp accelerates oxidation of ferrous ion to ferric ion.

Passive-gradient and facilitated-transport models of iron transport and kinetics in a macrophage cell culture incorporate chemical reactions in several domains. The chemical reactions occurring in these domains are shown in Table 2 for the passive-gradient model and Table 3 for the facilitated-transport model. The experimental system consists of a monolayer of cells in a culture dish in which the EC fluid is in contact with gas whose O_2_, N_2_ and CO_2_ fractions are fixed (Fig. 3). Whereas the two-domain passive-gradient model (Fig. 4) describes processes in intracellular (IC) and EC domains, the three-domain facilitated-transport model (Fig. 5A&B) distinguishes processes in IC, EC and (cellular) membrane domains. The SBGN process diagrams for the passive-gradient and facilitated-transport models are included as supplementary figures (Figs. S2 and S3). The IC and membrane domains are assumed to have uniform solute concentrations that vary only with time. For the IC domain (*I*), a dynamic mass balance leads to the time change of concentration of species (*j*):

**Table 2:** Reaction equations for passive gradient model.

Intracellular domain (I)	
**Species** 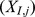	**Specific reaction rate** 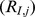
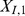 : Ferrous ion (Fe^2+^)	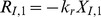 ; Intracellular oxidation ignored
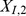 : Ferric ion (Fe^3+^)	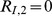 ; Intracellular oxidation ignored
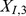 : Oxygen (O_2_)	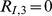 ; Intracellular oxidation ignored
**Extracellular domain (E)**	
**Species** 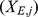	**Specific reaction rate** 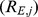
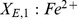	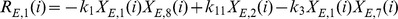
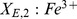	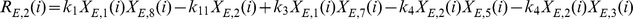
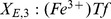	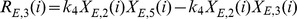
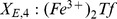	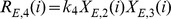
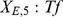	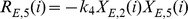
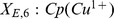	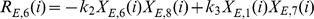
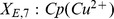	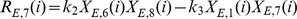
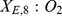	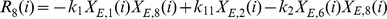

**Table 3:** Reaction equations for the facilitated-transport model.

Intracellular domain (I)	
**Species** 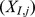	**Specific reaction rate** 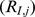
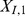 : Ferrous ion (Fe^2+^)	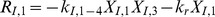
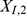 : Ferric ion (Fe^3+^)	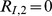 ; Intracellular oxidation ignored
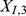 : Ferroportin (FPN)	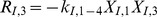
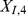 : Ferrous ion bound to FPN (Fe^2+^-FPN)	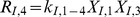
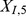 : Oxygen (O_2_)	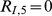 ; Intracellular oxidation ignored
**Membrane domain (M)**	
**Species** 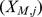	**Specific reaction rate** 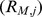
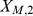 : Ferric ion (Fe^3+^)	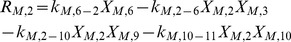
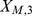 : Ferroportin (FPN)	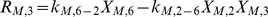
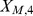 : Ferrous ion bound to FPN (Fe^2+^-FPN)	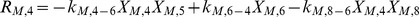
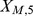 : Oxygen (O_2_)	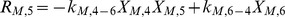
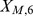 : Ferric ion bound to FPN (Fe^3+^-FPN)	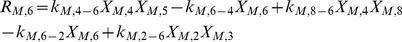
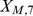 : Reduced Cp (Cp (Cu^1+^))	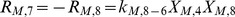
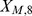 : Oxidized Cp (Cp (Cu^2+^))	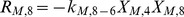
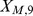 : Apo-Transferrin (Tf)	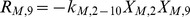
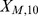 : Monoferric Transferrin (Fe^3+^)Tf	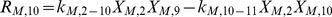
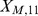 : Holo-transferrin (Fe^3+^)_2_Tf	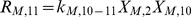
**Extracellular domain (E)**	
**Species** 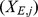	**Specific reaction rate** 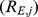
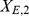 : Ferric ion (Fe^3+^)	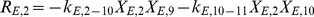
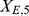 : Oxygen (O_2_)	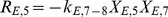
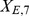 : Reduced Cp (Cp (Cu^1+^))	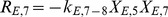
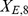 : Oxidized Cp (Cp (Cu^2+^))	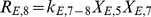
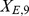 : Apo-Transferrin (Tf)	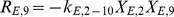
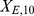 : Monoferric Transferrin (Fe^3+^)Tf	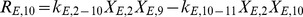
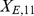 : Holo-transferrin (Fe^3+^)_2_Tf	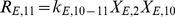

**Note:** We assume that both the binding sites on Tf are equivalent so that 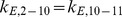 in the extra-cellular domain and also 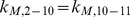 in the membrane domain.



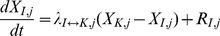
(5)Since the effective ratio of cellular domain area to volume cannot be clearly specified, these parameters are lumped into the mass transfer coefficient, which has the units of inverse time. Here, 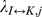 is a mass transport coefficient between domains *I* and *K* and 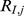 is the reaction rate corresponding to any solute 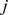 in the IC domain. In the two-domain passive-gradient models, transport occurs between the IC and EC (*K = E*) domains. In the three-domain model, transport occurs between the IC and membrane (*K = M*) domains and between the membrane and EC domains. This requires an additional set of equations for the membrane domain:
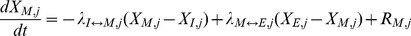
(6)


In the spatially lumped version of the passive-gradient model, the solute concentrations in the EC domain change only in time *X_j_(t*), not in space. In the spatially distributed models, solute concentrations in the EC domain change in time and in space *X_j_(z,t)*. The transport processes can be assumed to be one-dimensional in the direction perpendicular to the plane of the cells as the characteristic width of the system is much larger than the depth (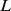) of the EC domain.
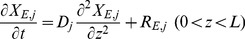
(7)where 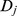 is the diffusion coefficient of solute *j*. At the closed interface of the EC fluid with the environment, the transport flux and, therefore, the concentration gradient is zero for all species except 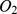. The 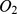 flux in the EC domain equals that from the environment into the EC domain:
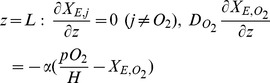
(8)where 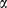 is the mass transport velocity (length/time) of oxygen. With spatially distributed diffusion in the EC domain of the passive-diffusion model, the IC-EC fluid boundary condition of the passive-diffusion model for any species 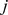 is



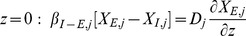
(9a)Similarly, for the facilitated-transport model, the membrane-EC domain boundary condition of the facilitated-transport model is
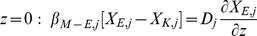
(9b)where 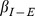 and 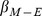 are solute mass transport velocities (length/time).

The cell culture system conserves the mass of iron (S = Fe), ceruloplasmin (S = Cp), and transferrin (S = Tf) in various forms. For the two-domain passive-gradient model, the total mass balance is
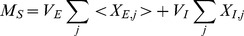
(10)


Here, we introduce the spatially averaged concentration in the EC domain:
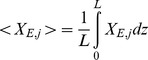
(11)


For the three-domain facilitated-transport model, the total mass balance is
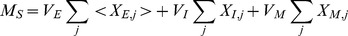
(12)where 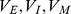 are the effective volumes of the domains.

#### Passive-gradient models

Fig. 4 incorporates the passive-gradient model diagram for either the spatially lumped (SL) or spatially distributed (SD) model. In both models, Fe^2+^ diffuses from the IC to the EC domain. In the EC, 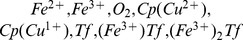 are involved in reactions as represented by kinetic equations (K.1)-(K.6) and in Table 1.

For the SL model, the iron diffusion rate from IC to EC is 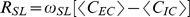
where 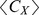 is the average iron concentration in the domain X = EC,IC and 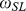 is the effective diffusion rate coefficient. For the SD model, the iron diffusion rate from IC to EC is
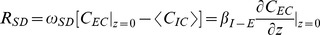
where 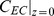 is the EC iron concentration at the cell membrane, 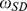 is the effective membrane transport rate coefficient and 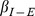 is ferrous mass transport velocity (length/time) and 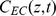 is the EC iron concentration at position z and time t.

In the IC domain, simultaneous dynamic processes of the mass transport of ferrous iron to EC domain occur along with sequestration of ferrous iron to ferritin nanocages in the IC domain. The sequestration is assumed to be a first-order reaction proportional to concentration of ferrous ion (shown by K.7a in facilitated-transport section). Between the IC and EC domains, this model assumes that FPN in the plasma membrane allows 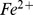 ions to diffuse freely. The cell membrane is assumed to be infinitesimally thin with reaction processes in the membrane ignored and the system is assumed to be one-dimensional in z-direction as shown in Fig. 3.

As represented by eq. (10), the total mass balances for iron, Cp and Tf in both domains are 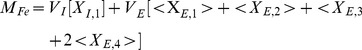
(13)




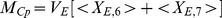
(14)




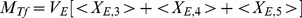
(15)


#### Facilitated-transport model

Within IC, membrane, and EC domains of the facilitated-transport model, the kinetics of the reaction processes (Fig. 5) are shown in Table 3. In the IC domain, the iron oxidation is neglected. The irreversible reaction of ferrous iron with ferritin in the IC assumes first-order removal of ferrous ions via sequestration to ferritin nanocages: 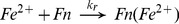
(K.7a)


Ferritin species with and without iron, 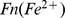 and (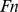) are assumed to be of secondary importance.

In the facilitated-transport mechanism, FPN plays an active role. FPN in the IC domain binds the ferrous iron as follows:
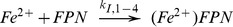
(K.7b)


While 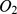 and FPN diffuse into the IC domain from the membrane, 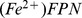 diffuses from the IC domain into the membrane. In the membrane domain, it reacts reversibly with oxygen:
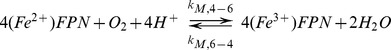
(K.8)


It is also oxidized by Cp having oxidized copper: 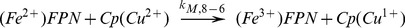
(K.9)


The oxidized 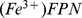 dissociates reversibly: 
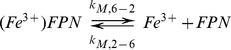
(K.10)


Binding of ferric ion to apo-Tf:
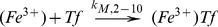
(K.11)


Binding of ferric ion to mono-ferric Tf to form holo-Tf:
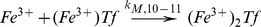
(K.12)


Whereas 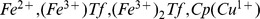 diffuse from the membrane into the EC space, 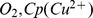 diffuse in the opposite direction. The three reactions in EC domain involving copper oxidation and binding of iron to transferrin (analogous to (K.3), (K.4), (K.5)) are:

Oxidation of reduced Cp: 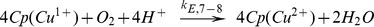
(K.13)


Binding of ferric ion to apo-Tf: 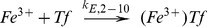
(K.14)


Binding of ferric ion to mono-ferric transferrin: 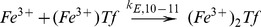
(K.15)


As represented by Eq. (12), total mass balances for iron, FPN, Cp, and Tf relate the concentrations in all domains:
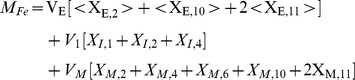
(16)




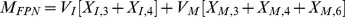
(17)




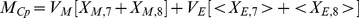
(18)




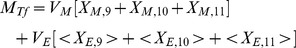
(19)


The mass balance for the ferric iron released from cells, which is compared between experimental data and simulated outputs, is
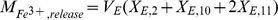



### Comparison of Models: Simulations and Data Analysis

To analyze data from the experiments in solution and cell culture, we applied computational models to simulate the experimental measurements under a variety of conditions with the same minimal set of parameter values. The initial conditions for these experiments are shown in Table 4 (for iron kinetics in solution) and Table 5 (for iron release in cell culture). The details related to the implementation of computational models to perform simulations depicting various experimental conditions are described in the methods section.

**Table 4:** Initial concentrations for simulations of iron kinetics in solution.

Simulation	Fe^2+^(μM)	Cp (μg/ml)	Apo-Tf (μM)	O_2_ (%)
No Cp	120	0	55	1
With Cp	120	120	55	1

**Table 5:** Initial concentrations for simulations of cellular iron release.

Simulation	Fe^2+^(μM)	Cp (μg/ml)	Apo-Tf (μM)	O_2_ (%)
No Cp	2.47	0	55	1
With Cp	2.47	300	55	1

#### Iron kinetics in solution

As specified in the methods section, kinetics of iron oxidation and binding to apo-Tf were studied in a well-mixed solution under many different conditions. The measured optical density (OD_460nm_) of ferric-transferrin as a function of time was related by linear calibration to the output, *y(t)*, that represents the concentrations of 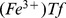 and 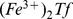.

The corresponding model output 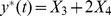 (according to stoichiometry of ferric ion) was computed by solving Eq. (1) with the reaction rates specified in Table 1 together with Eqs. (2)–(4) as needed.

**Figure 6:**
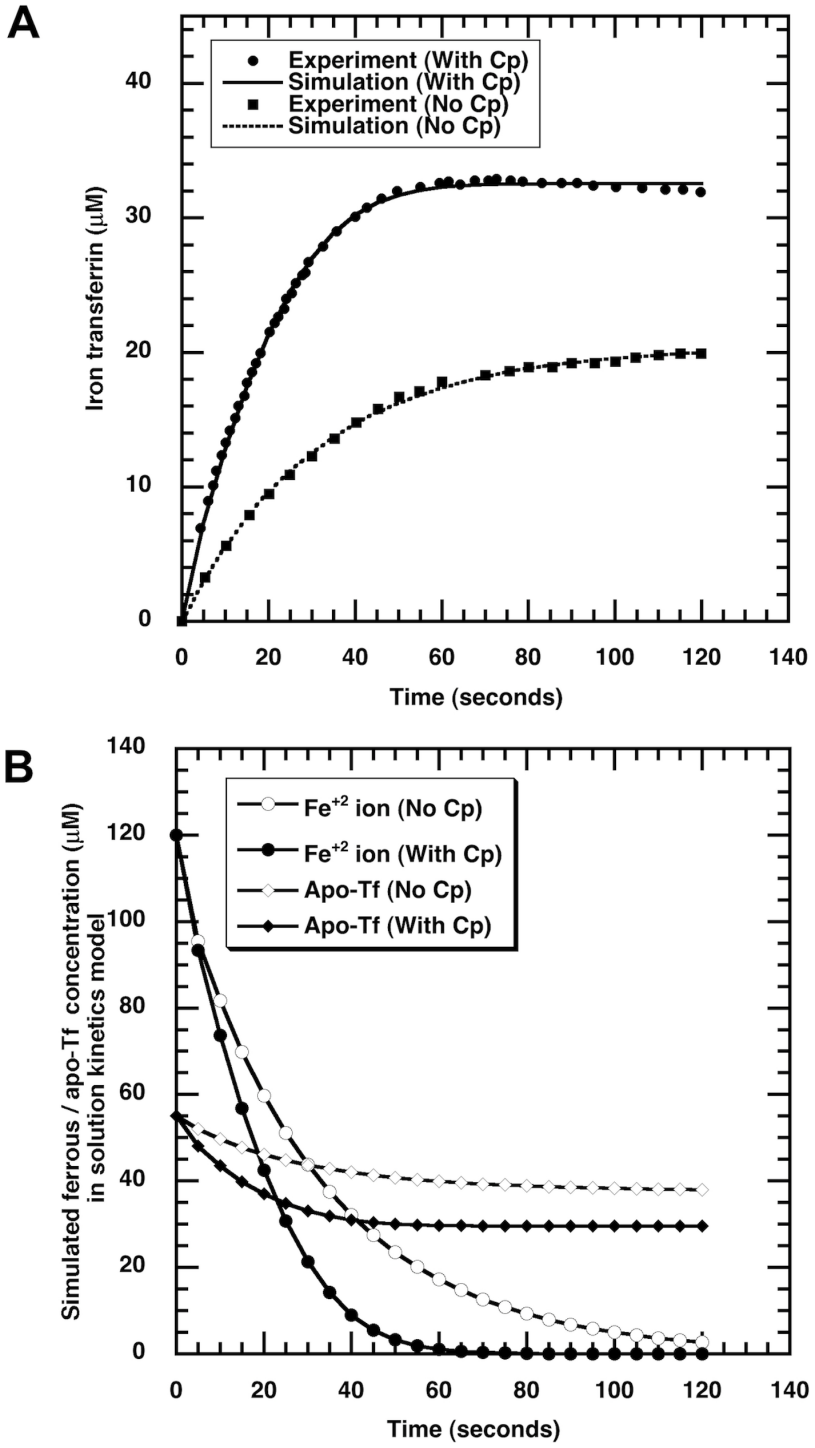
Simulation of iron kinetics in solution. Fig 6A: Comparison of experimental data with simulated output from solution kinetics model. Fig 6B: Simulated dynamics of ferrous iron and apo-Tf. With Cp, oxidation of ferrous iron as well as consumption of apo-Tf is faster.

The process of oxidation and binding of ferrous ion (Fe^2+^) to apo-Tf in a neutral pH solution was studied by measuring the dynamics of formation of mono- and diferric-Tf. The simulated output from the iron kinetics model closely matched the corresponding data for all experiments as illustrated in Fig. 6A. The diffusion coefficients of the various species and the optimal estimates of the parameters obtained during the simulations of iron kinetics in solution are shown in Table 6 and Table 7 respectively. In the absence of Cp, the rate of iron oxidation as well as the rate of iron binding to Tf is slower. In the presence of Cp, the oxidation of ferrous ions as well as consumption of apo-Tf is faster as evident from the simulated dynamics (Fig. 6B) of these species in first 20 seconds.

**Table 6:** Diffusion coefficients in solution.

Species	Diffusion coefficient (cm^2^/s)
O_2_	3.24e-6
Cp(Cu^2+^),Cp(Cu^1+^)	7.13e-9
Tf, (Fe^3+^)Tf, (Fe^3+^)_2_ Tf	9.02e-8
Fe^2+^, Fe^3+^	3.46e-6
FPN, FPN- Fe^2+^, FPN-Fe^3+^	3.46e-7

**Table 7:** Optimal estimates of parameters for iron kinetics in solution model.

Parameter (unit)	Value
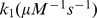	4.4e-3
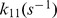	1.63e2
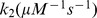	2.91
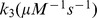	8.45
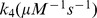	9.5e-3
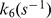	1.34

#### Iron release in cell culture

**Figure 7:**
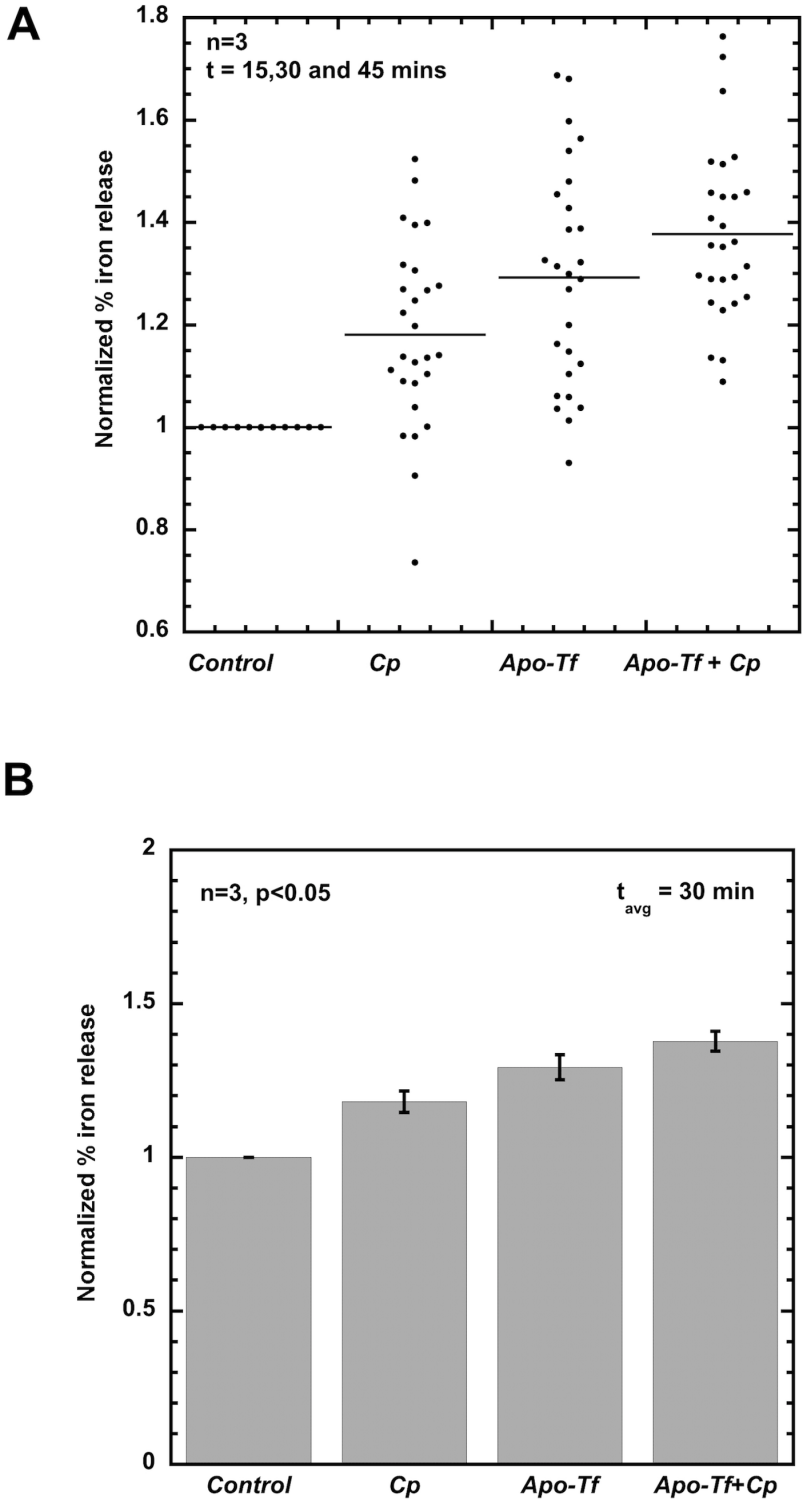
Experimental data of cellular iron release from macrophages in hypoxic environment under different conditions. Fig 7A: Normalized % iron release from three different experiments each with triplicates for mouse macrophages (RAW264.1 cell line) under hypoxic conditions (1% O_2_), Cp (300 µg/ml) and apo-Tf (55 µM) at 15, 30 and 45 min release intervals and at a given experimental condition (Control, Cp, apo-Tf and Cp plus apo-Tf). Each individual dot corresponds to normalized % iron release at a given release interval. Dots are grouped according to the experimental release condition indicated on x-axis. Fig 7B: Experimentally determined average normalized % iron release for each of the four experimental conditions (Control, Cp, apo-Tf, and Cp + apo-Tf) at 30 min using combined data from three experiments above. Error bars indicate standard error in all the data points for a given experimental condition. Iron release increased with statistical significance in the presence of Cp (p = 2.07e-5), apo-Tf (p = 1.53e-7) and apo-Tf + Cp (p = 8.7e-12). Iron release increased with statistical significance in the presence of apo-Tf + Cp relative to Cp (p = 6.5e-6) and apo-Tf (p = 6.8e-3).

In cell culture, the rate of iron release from macrophages can be quantified by the appearance of ferric ion in the medium (EC fluid) over time. As shown in Fig. 7, cellular iron release from the mouse macrophages (RAW264.1 cell line) is stimulated by Cp, apo-Tf and both together. In order to compare results of multiple experiments (with variations in iron uptake values), we expressed cellular iron release in terms of normalized % iron release. We calculated the % iron release under different conditions by dividing the release in that condition by the total uptake, so that uptake is 100%. The % iron release was then normalized with the control condition, that is, in presence of media only (Fig. 7A). In Fig. 7A, we plotted normalized % iron release from three different experiments each with triplicates for mouse macrophages (RAW264.1 cell line). These experiments were done under hypoxic conditions (1% O_2_) and release time intervals of either 15, 30 or 45 min at a given experimental condition (Control, Cp (300 µg/ml), apo-Tf (55 µM), and both Cp and apo-Tf). Each individual dot corresponds to normalized % iron release at any of the three release intervals. Dots are grouped according to the experimental release condition (Control, Cp, apo-Tf and both Cp and apo-Tf) as indicated on x-axis.

An average iron release over a time period of 15 to 45 minutes (average release time interval of 30 min) in the presence of Cp alone, apo-Tf alone and both Cp and apo-Tf was calculated by pooling the data points together from multiple experiments (Fig. 7B). Error bars indicate standard error in all the raw data (27 data points for each case) for a given experimental condition. A statistically significant enhancement in iron release was observed by addition of Cp (p = 2.07e-5), apo-Tf (p = 1.53e-7) and apo-Tf and Cp together (p = 8.7e-12) with respect to control. The stimulation in the presence of apo-Tf + Cp was significant with respect to Cp (p = 6.5e-6) alone and apo-Tf alone (p = 6.8e-3). This average experimental iron release at 30 min release interval was simulated in iron release models to perform static comparison of model outputs.

From cell culture studies under many different conditions, the iron release data reflects all the radioactive iron (^55^Fe) in the EC fluid as 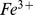, 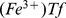, and 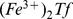. The molar quantity of the EC tracer iron is related to the scintillation count rate and efficiency. This, in turn, is related to the total molar quantity of iron *y(t)* by the specific activity. The corresponding model output is as follows:
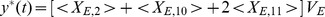
where *V_E_* is the EC fluid volume. This output was computed using both the passive-gradient (spatially lumped and distributed models) and facilitated-transport models.

#### Model comparisons

**Figure 8:**
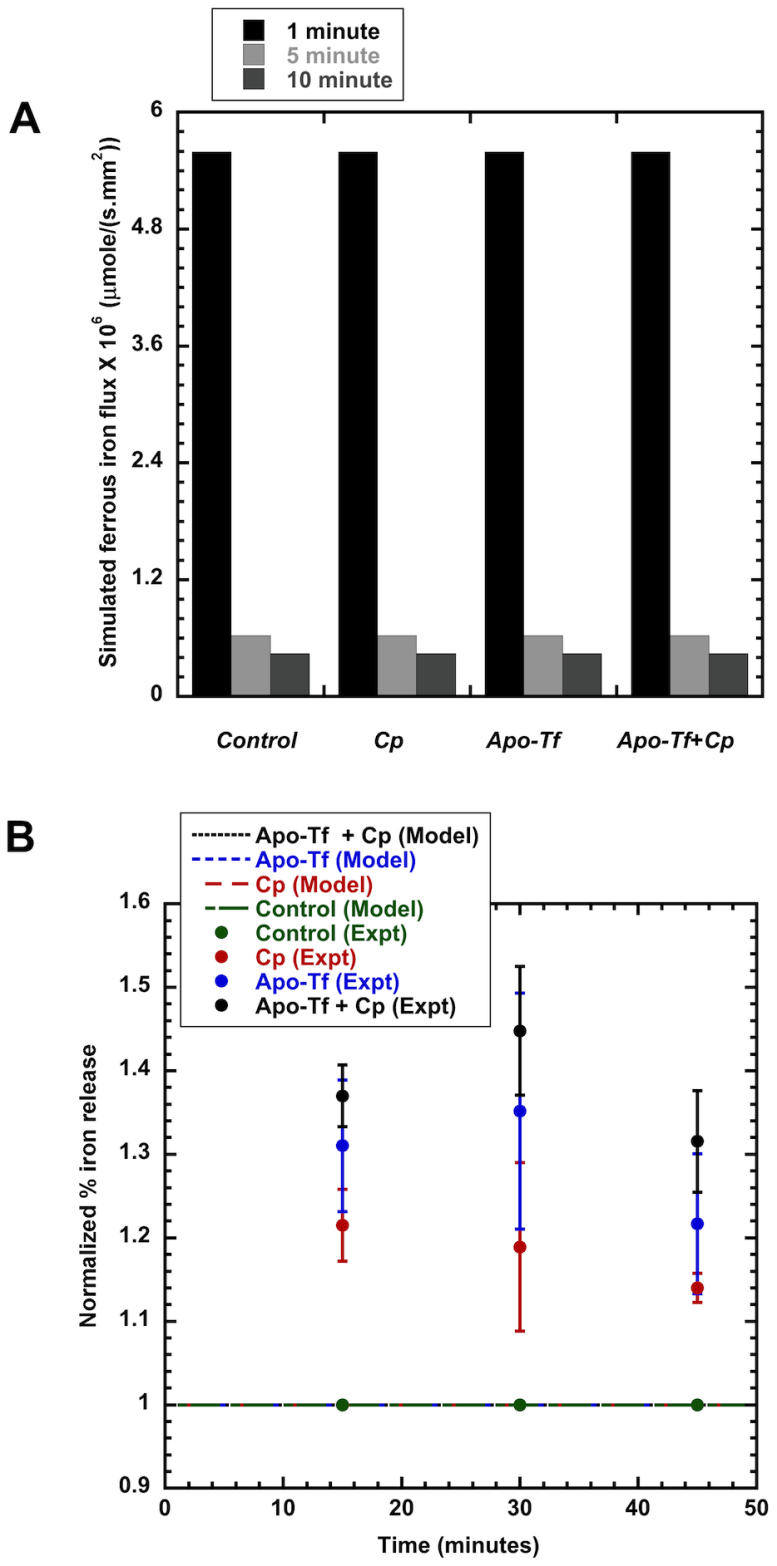
Simulation of passive-gradient models. Fig 8A: Simulated ferrous iron flux using passive-gradient SD model for the four extracellular medium conditions. Absolute values of simulated ferrous iron flux for passive-gradient model with addition of Cp (300 µg/ml), apo-Tf (55 µM) or apo-Tf + Cp in extracellular medium under hypoxic conditions (1% O_2_). Simulations show no flux change relative to control with addition of Cp, apo-Tf, or apo-Tf + Cp after 1, 5 or 10 min. Simulated flux dynamics decrease to the same extent with time for all four experimental conditions of extracellular medium. Fig 8B: Dynamic normalized % iron release from passive-gradient models. The output from both SL and SD models overlap with control. The passive-gradient models cannot simulate the enhanced iron release in the presence of Cp, apo-Tf and both.

With the passive-gradient model, optimal estimates of the parameters (Table 8) were obtained by fitting the model-simulated output to the iron-release data from the experiments without Cp and without apo-Tf. This model, however, could not fit all the data including the data from experiments with Cp, apo-Tf or apo-Tf + Cp with a single, consistent set of parameter values. To test the hypothesis that Cp, apo-Tf and apo-Tf + Cp alter the gradient for iron release as suggested by Osaki [5], the flux of iron across the cell membrane, 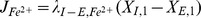, was evaluated at 1, 5 and 10 min for several conditions of the EC medium (Fig. 8A). The simulated flux of iron (rate/area) from IC to EC fluid decreased from 1 min to 10 min (Fig. 8A). However, neither the addition of Cp, apo-Tf nor apo-Tf with Cp changed the flux of iron over this period. We compared the dynamic normalized % iron release from passive-gradient models (SL and SD) with experimental data (Fig. 8B). None of the passive-gradient models could simulate the enhanced iron release in the presence of Cp, apo-Tf and both at any of the three release time points. The simulation trends from both models overlapped with the control normalized % iron release.

**Table 8:** Optimal estimates of parameters for passive-gradient model of iron release in cell culture.

Parameter	Value
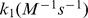	3.25e+1
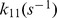	1.21e+2
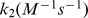	3.86
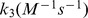	1.86e+1
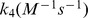	1.54e+1
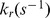	8.18e-2
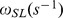	0.2e-5
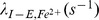	3.75e-5
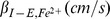	3.75e-5

**Figure 9:**
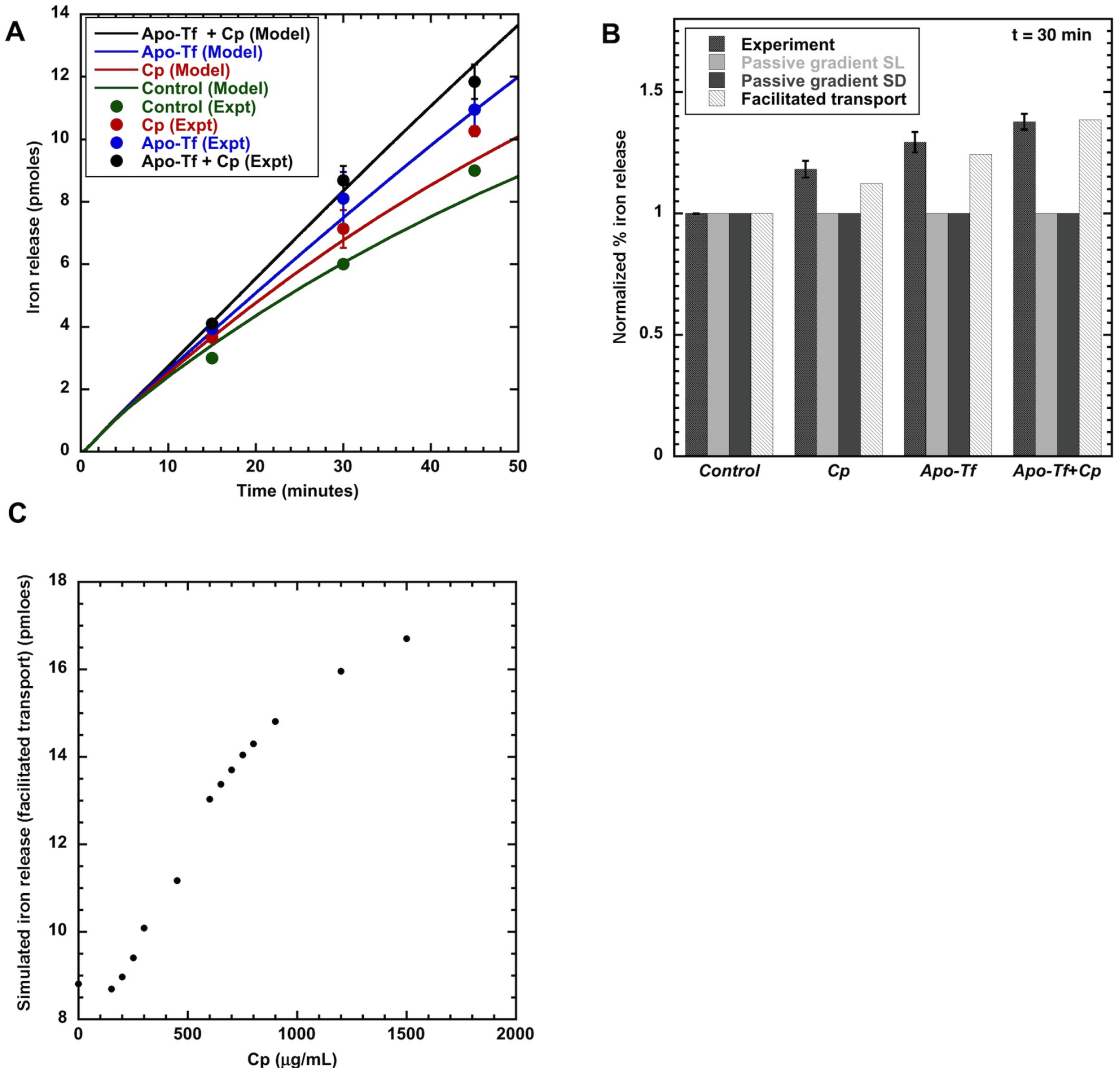
Simulated output from facilitated-transport model compared to experimental data. Fig. 9A: Facilitated-transport model simulation of the different experimental release conditions using the same model parameter values. Dynamic cellular iron release is compared from 0 to 50 min. Fig. 9B: Facilitated-transport model, but not passive-gradient model, simulates experimental increase in iron release with Cp (300 µg/ml), apo-Tf (55 µM) and apo-Tf + Cp under hypoxic conditions (1% O_2_). Static cellular iron release at average release time of 30 mins is compared. Fig. 9C: Facilitated-transport model simulates cellular iron release dependence on Cp.

With the facilitated-transport model, optimal estimates of the parameters (Table 9,10) were obtained by fitting the model-simulated dynamic responses to the dynamic iron-release data from all experiments over 45 min (Fig. 9A). From a single, consistent set of parameter values, the facilitated-transport model, but not the passive-gradient models, simulated the experimental data showing an increase in iron release at 30 min release interval with Cp, apo-Tf or apo-Tf + Cp (Fig. 9B). Model simulations with both forms of passive-gradient models (SL and SD) could not reproduce the experimental data showing an increase in iron release with apo-Tf or apo-Tf + Cp (Fig. 9B). The change of cellular iron release with a change in Cp concentration is predicted by the facilitated-transport model simulation (Fig. 9C). This model provides a mechanism consistent with the experimental observation of decreased iron release at lower Cp concentration [10].

**Table 9:** Optimal estimates of reaction rate coefficients for facilitated-transport model of iron release in cell culture.

Parameter	Value
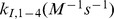	8.8 e+1
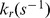	8.18e-2
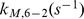	3.4
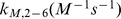	1.72
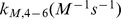	3.25e+1
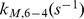	1.21e+2
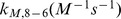	3.86
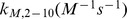 = 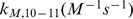	1.54e+1
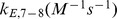	1.86e+1
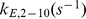 = 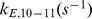	1.54e+1

**Table 10:** Optimal estimates of species transport coefficients for facilitated-transport model of iron release in cell culture.

Parameter	Value*	Associated species
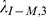	0.297	Ferroportin (FPN)
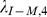	0.01	Ferrous ion bound to FPN (Fe^2+^-FPN)
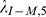	0.00391	Oxygen (O_2_)
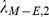	0.09	Ferric ion (Fe^3+^)
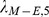	0.00391	Oxygen (O_2_)
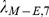	0.0329	Reduced Cp (Cp (Cu^1+^))
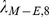	0.0329	Oxidized Cp (Cp (Cu^2+^))
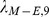	0.277	Apo-Transferrin (Tf)
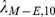	0.277	Monoferric Transferrin (Fe^3+^)Tf
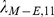	0.277	Holo-transferrin (Fe^3+^)_2_Tf
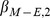	0.09	Ferric ion (Fe^3+^)
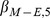	0.00391	Oxygen (O_2_)
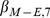	0.0329	Reduced Cp (Cp (Cu^1+^))
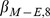	0.0329	Oxidized Cp (Cp (Cu^2+^))
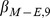	0.277	Apo-Transferrin (Tf)
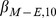	0.277	Monoferric Transferrin (Fe^3+^)Tf
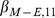	0.277	Holo-transferrin (Fe^3+^)_2_Tf

* Unit of 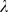 's in s^−1^ and 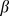 's in cm/s.

#### Quantifying a key transport mechanism

**Figure 10:**
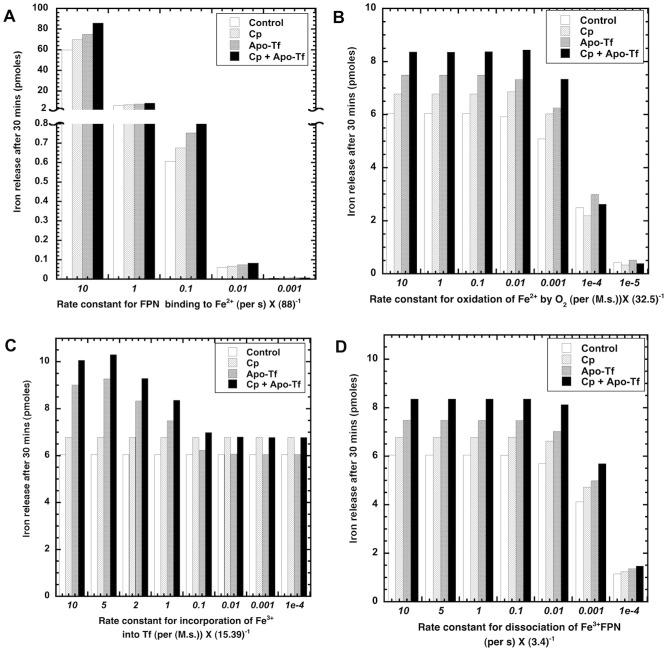
Sensitivity analysis of iron release using facilitated-transport model parameters. Fig 10A: Simulation with facilitated-transport model quantifies the sensitivity of iron release to changes in key model rate parameter (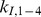) that determines transport of ferrous ion by binding to FPN with all other parameters constant. Sensitivity of cellular iron release to rate constant for oxidation of ferrous by oxygen (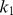) (Fig. 10B), rate constant for incorporation of ferric iron into Tf (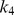) (Fig. 10C) and rate constant for forward dissociation of ferric-FPN (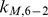) (Fig. 10D) is also shown here.

The relative importance of any process contributing to iron release can be evaluated by computing the sensitivity of the output to change of the corresponding parameter value. Here, we examined the change of the total concentration of iron in the EC fluid in response to the change in the parameter associated with this process. The key parameter associated with facilitated-transport mechanism distinguishing it from the passive-gradient mechanism is the binding of ferrous ion to FPN. Hence we studied the response of the system (iron release in EC medium) in response to order of magnitude changes in the parameter related to the binding of ferrous iron to FPN (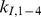) by keeping all other parameters constant. We also repeated sensitivity analysis with some other key parameters as follows: rate constant for oxidation of ferrous by oxygen (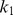), rate constant for incorporation of ferric iron into Tf (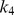) and rate constant for forward dissociation of ferric-FPN (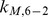). This is shown as different panels in Fig. 10.

Sensitivity of iron release to a change in the binding of ferrous ion and FPN is revealed by simulating the response to changes in the parameter 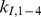 of the facilitated-transport model. Simulations show that an increase or decrease in the parameter 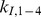 directly impacts the cellular iron release (Fig. 10A). Cellular iron release is found to be more sensitive to rate constant for binding of ferrous iron to FPN compared to other rate constants. Sensitivity of cellular iron release is shown for oxidation of ferrous by oxygen (Fig. 10B), incorporation of ferric iron into Tf (Fig. 10C) and rate constant for forward dissociation of ferric-FPN (Fig. 10D). This analysis quantifies the binding of ferrous ion to FPN as the key process driving the transport of ferrous iron to the membrane. It is postulated that FPN carries the bound ferrous ion to cellular membrane via vesicular transport.

## Discussion

### Mathematical Models to Quantify and Distinguish Transport Mechanisms

Iron release from macrophages, which is one of the most important processes involved in control of iron homeostasis, has been assumed to follow a passive-diffusion model. The “gradient hypothesis” associated with this passive-diffusion model was developed for gut iron absorption by Saltman [2] and supported by Osaki [5], [20], but not analyzed mathematically. In this study, we developed and compared passive-gradient and facilitated-transport models to explain iron release from macrophages.

The presence of Cp, the multi-copper ferroxidase, catalyzes the oxidation of ferrous ion, which is consistent with model simulations. The consumption of apo-Tf for ferric iron incorporation is also accelerated in the presence of Cp (Fig. 6B). These observations confirm the catalyzing ability of Cp. Our studies indicate that the passive-gradient hypothesis [5] proposed to describe the stimulation of iron release by Cp from the gut cannot explain the iron efflux mechanism from macrophages (Fig. 9B). Although some experimental observations might suggest the efflux mechanism conforms to the gradient hypothesis, mathematical analysis and simulations of data with passive-gradient models do not support this hypothesis. Theoretically, ferrous ions as they diffuse to the cell membrane are quickly oxidized making the concentration of ferrous iron just outside the cell membrane negligible. Addition of Cp or apo-Tf or both cannot significantly reduce the already negligible ferrous ion concentration. No change in the flux of iron occurs in simulations of the passive-gradient model under these experimental conditions (Fig. 8A). Consequently, the passive-gradient model cannot explain the stimulation of iron release from macrophages.

The facilitated-transport model with a single, optimal set of parameter values simulated all the iron-release data (Fig. 9B). The parameter values obtained initially from *in vitro* kinetics in solution did not change significantly from the values found from cellular experiments. Based on simulations, the facilitated-transport model indicates that Cp acts as a ferroxidase to increase iron release from macrophages in the presence of apo-Tf under physiological conditions of O_2_. Experimentally, maximal iron release from macrophages was observed under hypoxia in the presence of apo-Tf and Cp together. Simulations with the facilitated-transport model indicate that decreased Cp concentration results in decreased iron release from macrophage cells leading to possible iron retention in cells (Fig. 9C). With the facilitated-transport model, it is evident that Cp deficiency can cause iron overload diseases through retention of iron in macrophage cells.

The main difference between the passive-gradient models (SL and SD) and the facilitated-transport model is the effect of FPN-mediated facilitated-transport of iron to the plasma membrane (Fig. 1). Instead of a passive channel, FPN plays an active role in iron efflux in the facilitated-transport mechanism. While there have been reports of iron transport being a facilitated-transport process [21], this mechanism had not been quantitatively tested using a mathematical model. Simulations using mathematical model based on facilitated-transport reveal that iron release is sensitive to the reaction rate coefficient for binding FPN and ferrous ion in all the four experimental conditions of extracellular medium. Furthermore, the facilitated-transport of iron by FPN to the plasma membrane provides a mechanism of stimulated iron release by Cp, apo-Tf, or apo-Tf + Cp.

### Model Assumptions and Limitations

Because of unavailability of data regarding concentration of FPN molecules in the intracellular domain, we assumed its concentration to be at least twice the concentration of ferrous iron. Although FPN is assumed to be in excess of ferrous ion to facilitate its transport across the cell, this assumption does not affect model outcomes. It is known that FPN is expressed in intracellular vesicles and can get transported to plasma membrane of macrophages [22]. This information was incorporated into the model. The FPN in the membrane compartment is internalized and transported back to the cell as soon as ferric-FPN dissociates. The diffusion parameters for the transport of FPN between the IC and the membrane domains (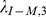 and 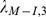) were assumed to be identical. Inside a cell, the processes of vesicular trafficking to the plasma membrane and endocytosis are quite separate and the kinetics may differ. This distinction could be made if there were experimental data on the kinetics of the exocytosis and endocytosis of FPN. Although the iron binding sites of apo-Tf may differ to some extent, the model assumes them to be identical. Analysis of the solution kinetic model shows that this assumption does not limit the ability of the model to predict the experimental results. Furthermore, this assumption would not significantly alter simulations of the cellular processes by the facilitated-transport model. In the experimental studies in which apo-Tf was added to the EC domain, we assume that the consequent mono-ferric and di-ferric Tf species remain in the EC fluid. However, both apo- and holo-Tf could be taken up by the cells through Tf receptor endocytosis. Hence, the facilitated transport model in its current form may not correctly predict iron release at longer release interval in hours.

### Future Directions

The facilitated-transport model can be the basis for developing other physiologically relevant models of iron release that incorporate important proteins such as hepcidin [23]. Although iron release from macrophages is very tightly controlled through control of FPN expression, this is not addressed in the current model. This aspect could be incorporated by considering the change of gene expression of FPN as well as protein degradation through interaction with hepcidin. From a systems perspective of the control of iron release from macrophages, control loops and interactions can be added to the present model. Furthermore, the facilitated-transport model iron release mechanism can be included in a whole-body model of iron metabolism to predict more accurate iron status in body. This would be important for devising patient-specific treatments in chronic kidney disease.

## Methods

### Experimental Studies

#### Iron kinetics in solution

The kinetics of iron oxidation and binding to apo-transferrin was studied in a well-mixed solution by the spectroscopic measurement of formation of ferric transferrin (OD_460nm_) using a modification of the method of Osaki et al. (5). Ferrous ammonium sulfate was dissolved in glycine (0.1 mM) buffer at pH 3.0. Apo-transferrin and Cp were dissolved in RPMI 1640 medium (without phenol-red dye) containing 20 mM HEPES at pH 7.4. Apo-transferrin was used at 55 µM, the upper limit of the normal range in adult serum. For these experiments, the solutions contained 1% O_2_, 5% CO_2_, and 94% N_2_ by bubbling with a mixture of N_2_, CO_2_, and O_2_ at the appropriate ratios using a 3-channel gas controller and mixer. In some experiments, O_2_ or CO_2_ concentration was varied independently. For each experiment, a solution of all components except iron was placed in a plastic cuvette. The reaction process began when a ferrous ion solution was added to the cuvette to make a total volume of 1 ml. The cuvette was sealed with parafilm and transferrin-bound iron was measured as OD 460 nm at 2 sec intervals.

Purified human Cp was obtained from Vital Products (Boynton Beach, FL) and purity was verified by an absorbance ratio (610 nm/280 nm) greater than 0.04. Integrity of the protein was established by SDS-PAGE and Coomassie stain; intact, 132 kDa protein was the predominant form, and the primary degradation product was the 115 kDa protein present in human serum. Cp mass was determined by nephelometry by the Reference Laboratory at the Cleveland Clinic Foundation, and metal content was determined by inductively-coupled plasma mass spectroscopy. Cp contained 6.3±0.3 copper atoms per molecule, and the iron content was below the instrument detection level (i.e., <0.1 atoms per molecule). Human apo-transferrin was obtained from Calbiochem (Billerica, MA). The iron content of apo-transferrin was 0.060±0.002 atoms per molecule; the copper content of the transferrin preparations was negligible (i.e., <0.001 atoms per molecule). Ferrous ammonium sulphate was from Sigma (St. Louis, MO).

#### Iron release from macrophages

Iron release from macrophages in a cell culture was quantified by measurement of ^55^Fe in the extracellular medium after loading cells with ^55^Fe-NTA (nitrilotriacetic acid). The experiment was repeated for uptake time of 3 h, constant O_2_ levels of 1% in the environment, and Cp concentrations of 300 µg/ml in the medium. Mouse macrophages (RAW 264.7 cells) were grown to a density of about 1×10^6^ cells/ml in RPMI-1640 medium containing 10% fetal bovine serum. The cells were centrifuged at 1,000×g for 5 min, the supernatant aspirated, and the pellet washed with phosphate-buffered saline (PBS). The cells were plated at density of 0.5×10^5^ cells/well in a 12-well plate. To load the cells with iron, the media and non-adherent cells were aspirated, and the adherent cells were washed and incubated in serum-free RPMI 1640 medium for 2 h. The cells were incubated with ^55^Fe-NTA (10 µM) in the same medium containing ascorbate (100 µM) for 3 h in a hypoxia chamber (Pro-Ox, Reming, Redfield, NY). When incubated for up to 3 h, intracellular iron remained in the labile form (Fe^2+^).

Ascorbate has been shown to enhance mobilization of Fe^2+^ from ferritin and tissue deposits, as it is a reducing agent as well an iron chelator. During iron loading for 3 hours, in our experiments, ascorbate was used for the same purpose (that is, to keep iron in the reduced form and consequently become more effectively available for cellular release at the membrane where Cp exerts its ferroxidase activity). In our experiments, we have exposed the cells with ascorbate containing medium for 3 hr, during which cell viability shouldn't be adversely affected. Indeed, treatment duration as long as 24–72 hours has been kept by several groups for observing the relevant effect on cell survival [24]. Also, ascorbate concentration used in our experiments was 100 µM. Since physiological concentration of ascorbate in the monocytes is generally around 350–400 µM, this process is unlikely to contribute to pathological oxidation reactions [25].

The chamber was maintained at 37 °C with an atmosphere regulated to contain 1% O_2_ using an oxygen controller (Pro-Ox model 110, Reming), and with the remainder occupied by a mixture of 5% CO_2_ and 95% N_2_. Cell viability after 3 h of hypoxia was determined by trypan blue dye exclusion. For experiment validity, consistent cell viability greater than 95% was required under all conditions. The medium was aspirated and the cells were washed with ice-cold PBS containing 100 µM EDTA to remove iron nonspecifically bound to the cell surface, and twice with ice-cold PBS. ^55^Fe-NTA uptake was measured in individual wells by lysis in 20% NP-40 followed by liquid scintillation counting.

Cells were treated with Fe-NTA for iron loading following which cellular iron release was observed. Treatment with Fe-NTA complex is a standard procedure to load the cells with non-transferrin bound iron (NTBI) and used commonly in the field for *in vitro* and animal studies. *In vitro* studies report that Fe-NTA is taken up more efficiently than transferrin-bound iron (TBI) making the use of Fe-NTA as the method choice for experimental iron loading of cells [26], [27]. Note that TBI was not used for cellular iron loading because recycling of apo-Tf to the cell membrane would confound the latter part of the experiment (that is, iron release using apo-Tf only or apo-Tf + Cp).

To measure ^55^Fe release, test reagents (apo-Tf, Cp) were dissolved in serum-free RPMI-1640 medium containing 20 mM HEPES at pH 7.4, and the solutions exhaustively bubbled with gases at the appropriate O_2_ and CO_2_ concentrations before addition to cells. The cells loaded with ^55^Fe were washed with cold PBS and placed in the new medium in the hypoxia chamber for either 15 or 30 or 45-min iron release interval. The medium was collected and counted by liquid scintillation.

Apo-Tf and Cp were obtained as described above in the kinetic studies in solution. RPMI-1640 medium (Invitrogen, Carlsbad, CA) was selected for cell experiments since it does not contain iron, copper, or ascorbic acid, a metal ion reducing agent. NTA, ferric chloride and all other reagents were from Sigma (St. Louis, MO). ^55^FeCl_3_ (9.1 mM, 20 mCi/mg) was obtained from Perkin Elmer. A ^55^Fe-NTA solution was prepared by incubating ^55^FeCl_3_ (9.1 mM) and NTA (45.5 mM) for 1 h. The resulting ^55^Fe-NTA solution was mixed at a 1∶9 molar ratio with unlabeled Fe-NTA (prepared similarly) in serum-free medium containing ascorbate (100 µM) so that the final concentration of ^55^Fe-NTA was 10 µM. Iron solutions were freshly prepared before each experiment.

### Simulation Details

The model parameters belong to the following categories: reaction rate coefficients, transport coefficients and initial concentrations (associated with each experimental condition). For the spatially distributed models, the spatial derivatives were discretized (i.e., method of lines [28]) so that in all cases, the models had the form of a set of ordinary, first-order differential equations. These models expressed as initial-value problems were solved numerically with a stiff integrator, ‘ode15s’ (MATLAB, MathWorks Inc.). The values of some parameters are known by direct measurements and others were obtained from the literature. Most parameter values (viz., reaction rate and transport coefficients) were estimated by least-squares fitting of model simulated outputs to experimental data at many time points (*t_i_: i = 1, 2,…n*). This was accomplished by non-linear optimization with constraints implemented using ‘lsqcurvefit’ (MATLAB, MathWorks Inc.). The estimation procedure was repeated with different initial parameter values to show convergence to global optimal values. A sequential process was followed. First, data from the experiments in solution were simulated using the corresponding iron kinetics model. Then, the passive-gradient and facilitated-transport models were tested to determine if these could simulate data from cell culture experiments with optimal parameter estimates. We rejected a model if it could not simulate all the data satisfactorily with the same parameter values.

We used lsqcurvefit, which is a gradient-based algorithm that incorporates constraints as lower and upper bounds on the parameter values. This greatly improves its efficiency in minimizing the least-squares objective function. We used lsqcurvefit for optimal estimation because it is faster than other optimization algorithms such as genetic algorithm. This algorithm has been routinely used by scientific community [29], [30]. With the experimental data available, model parameters can be estimated more efficiently in a step-wise process. First, we estimated parameters with a subset model that describes solution kinetics. The parameter values from this model were applied in estimating parameters of the more complex passive and facilitated- transport models. With this procedure, fewer parameters with no prior knowledge are estimated on each step to provide better precision and accuracy of the estimates. This step-wise approach has been used previously with demonstrated success in even more challenging applications [31].

For each of the four models (solution kinetics, passive-gradient SL, passive-gradient SD and facilitated-transport), we estimated parameter values that provided the best fit of the model output to the experimental data in the following sequence: first, iron release in the medium only (control), then in the presence of apo-Tf alone, Cp alone, and both Cp and apo-Tf. The iron release models were simulated by starting with a reduced model that did not consist of any species related to Cp or apo-Tf and devoid of any processes related to those species. The reduced model with iron release in the medium alone does not contain reaction process represented by equations K.2, K.3, K.4, K.5, K.9, K.11, K.12, K.13, K.14 and K.15 (that is, involving either Cp and apo-Tf). The reduced model with iron release in the presence of apo-Tf alone excluded any reaction process involving Cp, while the one with iron release in the presence of Cp alone excluded reaction processes involving apo-Tf. We have specified each chemical species (j) involved in a given reduced version of model during stage-wise parameter estimation.

We recreated the model process diagrams in SBGN graphical notation (made in Cell Designer) and have provided them as supplementary figures along with the MATLAB codes (Data S1) used to implement each model. We wrote custom MATLAB codes to run the models instead of using ordinary differential equation (ODE) solver in SBML software (Cell Designer). This is easier for use with the method of lines that converts partial differential equations in some of our models to ODEs. Electronic formats of our models (MATLAB codes) along with experimental data used to fit the models are supplied as supplementary information (Data S1).

#### Iron kinetics in solution

The measured optical density (OD_460nm_) of ferric-transferrin as a function of time was related by linear calibration to the output, *y(t)*, that represents the concentrations of 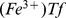 and 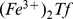. The corresponding model output 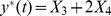 (according to stoichiometry of ferric ion) was computed by solving Eq. (1) with the reaction rates specified in Table 1 together with Eqs. (2)-(4) as needed. The following analysis sequence was used:

1. To simulate data from experiments without Cp, we used a reduced model with eq. (1) for j = 1,2,3,4,5,8 that involve independent rate coefficients 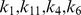. The two iron binding sites of apo-Tf are identical, 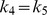.
2. To simulate data from experiments with Cp, the model consisted of Eq. (1) for j = 1, 2,..,8 together with Eqs. (2), (3), and (4). Here, we used values of 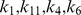 from step 1 and estimated only two unknown rate coefficients 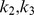.
3. To verify overall consistency of the parameter values, we re-estimated these 6 parameters simultaneously using data from all experiments (Table 7) and the parameter values from steps 1 and 2 as initial guesses.

#### Iron release in cell culture

From cell culture studies under many different conditions, the iron release data reflects all the radioactive iron (^55^Fe) in the extracellular fluid as 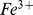, 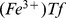, and 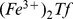. The molar quantity of the extracellular tracer iron is related to the scintillation count rate and efficiency. This, in turn, is related to the total molar quantity of iron *y(t)* by the specific activity. The corresponding model output is as follows:
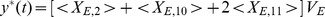
where *V_E_* is the extracellular fluid volume. This output was computed using both the passive-gradient (spatially lumped and distributed models) and facilitated-transport models.

The diffusion coefficients (Table 6) for O_2_ and iron were obtained from the literature [32]. The diffusion coefficients for the other molecules were estimated on the basis of molecular weight according to the inverse square-root relationship [32].

For the passive-gradient model, the initial values of the reaction rate coefficients were assumed to be the same as those for the iron kinetics model in solution. Optimal estimates of these coefficients and the mass transfer rate for ferrous ion (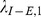) from IC to EC domain were obtained. For the facilitated-transport model, some reactions are the same as those for the iron kinetics model in solution. Therefore, the values of the reaction rate coefficients in these reactions were initial values for the optimal re-estimation of corresponding reaction rate coefficients:


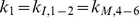
, 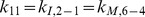, 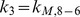, 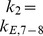, 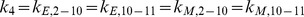


We assumed that the transport coefficients for similar chemical species were identical: 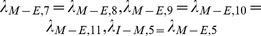. Also, diffusion coefficient for FPN and all associated species was assumed to be 1/10^th^ of that of ferrous species.

Then, a sequential estimation process was applied to get the optimal estimates of all parameters by matching model-simulated outputs with experimental data in the following sequence:

1. Using the above-mentioned values, we estimated 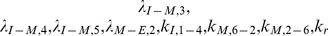. This required matching the data for experiments without apo-Tf with a corresponding reduced model (that is with iron release in medium alone which has been described in a previous section) having a reduced set of equations and parameters.
2. Using all the above parameter values as prior information, we estimated the remaining parameters 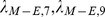 using the data sets: apo-Tf without Cp and apo-Tf with Cp.
3. The following parameters were re-estimated by taking above values as initial values for performing ‘lsqcurvefit’ to determine optimal parameter set by fitting to all data: 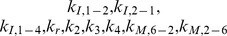

## Supporting Information

Figure S1 **Systems biology graphical notation (SBGN) process diagram for solution kinetics model.**
(EPS)

Figure S2 **SBGN process diagram for passive-gradient models.**
(EPS)

Figure S3 **SBGN process diagram for facilitated-transport model.** Abbreviations: Ferrous iron (Fe^2+^), Ferric iron (Fe^3+^), Ferroportin (FPN), Ferrous ion bound to FPN (Fe^2+^-FPN), Oxygen (O_2_), Reduced Cp (Cp (Cu^1+^)), Oxidized Cp (Cp (Cu^2+^)), Apo-Transferrin (Tf), Monoferric Transferrin (Fe^3+^)Tf, Holo-transferrin (Fe^3+^)_2_Tf.
(EPS)

Data S1 SolKinNoCp.m in Data S1: Matlab code for simulation of solution kinetics model using experimental data without Cp. SolKinWithCp.m in Data S1: Matlab code for simulation of solution kinetics model using experimental data in the presence of Cp. SolKinAllData.m in Data S1: Matlab code for simulation of solution kinetics model using the full experimental data (with and without Cp). xdata_nocp.mat in Data S1: Matlab input file for codes SolKinNoCp.m and SolKinAllData.m ydata_nocp.mat in Data S1: Matlab input file for codes SolKinNoCp.m and SolKinAllData.m x_new_withcp.mat in Data S1: Matlab input file for codes SolKinWithCp.m and SolKinAllData.m y_new_withcp.mat in Data S1: Matlab input file for codes SolKinWithCp.m and SolKinAllData.m ExperimentalData.xls in Data S1: Experimental data for fitting the three cellular iron release models. passive_lumped.m in Data S1: Matlab code for simulation of passive-gradient spatially lumped model. passive_model_distributed.m in Data S1: Matlab code for simulation of passive-gradient spatially distributed model. facilitated_model.m in Data S1: Matlab code for simulation of facilitated-transport model. dss044.m in Data S1: Matlab subroutine to implement method of lines to convert partial differential equations to ordinary differential equations in spatially distributed form of models (passive-gradient SD and facilitated-transport models).
(ZIP)
